# Advances of Laparoscopy for the Diagnosis of Pelvic Congestion Syndrome

**DOI:** 10.3390/medicina57101041

**Published:** 2021-09-30

**Authors:** Christos Arnaoutoglou, Rita S. Variawa, Paul Zarogoulidis, Aris Ioannidis, Nikolaos Machairiotis

**Affiliations:** 1 1st Department of Obstetrics & Gynecology, Papageorgiou Hospital, Aristotle University of Thessaloniki, 57001 Thessaloniki, Greece; 2 Independent Pharmacovigilance (Evaluation & Risk Management) Scientist, London E14 4HB, UK; rita.variawa@hotmail.com; 3 3rd Surgery Department, AHEPA University General Hospital, Aristotle University of Thessaloniki, 57001 Thessaloniki, Greece; pzarog@hotmail.com; 4 Surgery Department, “Genesis” Private Clinic, 57001 Thessaloniki, Greece; ariioann@yahoo.gr; 5 Fellow in Endometriosis and Minimal Access Surgery, Northwick Park, Central Middlesex and Ealing Hospitals, Acton Ln, London NW10 7NS, UK; nikolaosmachairiotis@gmail.com; 6 London North West University Healthcare NHS Trust, London HA1 3UJ, UK

**Keywords:** chronic pelvic pain, pelvic congestion syndrome, laparoscopy

## Abstract

The objective of this review is to describe the effectiveness of laparoscopy in the diagnosis and treatment of pelvic congestion syndrome (PCS). PCS is a cause of chronic pelvic pain (CPP) and is associated with dysfunction of the pelvic venous system. PCS is more common in women of reproductive age, and hormonal changes are associated with its development along with other reasons (e.g., working and living habits). There is an urgent need to establish an effective algorithm for the diagnosis and treatment of CPP, which could have a dramatic effect in patients’ everyday life. This algorithm should be able to overcome known issues that lead to the underdiagnosis of PCS, such as the overlap of its symptoms with other diseases. Here, we present our findings from literature articles about the methods used in practice today for the diagnosis of this syndrome. We also compare the methods to propose the most promising technique for providing a diagnosis with high accuracy. In our understanding, laparoscopy is superior when compared to other methods. It can provide a diagnosis of PCS while excluding or identifying other comorbidities and can also lead toward the next steps for the treatment of PCS.

## 1. Introduction

### Pelvic Congestion Syndrome

Pelvic congestion syndrome (PCS) is a condition that refers to dysfunction of the pelvic venous system. The cause of this condition is pelvic vein insufficiency, which results in the abnormal dilation of the interlinked venous channels of the ovarian veins and the internal iliac veins. Both the internal iliac and ovarian veins systems run through the broad ligament. Pelvic varices occur in the ovarian veins in most of the cases and 60% of these are diagnosed with PCS. In these cases, incompetency of the internal pudendal and broad ligament parametrial branches has been reported [1].

This incompetency mentioned has two primary causes. First, it may arise due to the absence of the venous valves or the incompetency of the valves. This may be associated with the alteration of any characteristic of the valvular system that causes its failure (e.g., elasticity of the vein wall, valvular dilation, venous valves). This dysfunction results in retrograde flow, slow blood flow (congestion), and reflux [2]. Second, the external compression of the vein that causes venous outflow obstruction may contribute to the development of the incompetency. Syndromes that promote the external compression are May–Thurner syndrome (associates with left common iliac vein) and nutcracker syndrome (associates with the left renal vein), which may have similar symptoms. Usually, PCS refers to ovarian veins and lower extremity pelvic pain.

The occurrence of PCS is more common in women of reproductive age (20–45 years) and no evidence of the condition has been observed in menopausal women. Epidemiology data of the syndrome are limited, as its primary symptom is chronic pelvic pain (CPP) and PCS is usually underestimated. So far, we know that 30% of the patients with CPP suffer from PCS [3].

The likelihood of PCS is increased with the number of pregnancies, and this may be explained by the hormonal changes occurring during pregnancy, which effect ovarian venous dilation. In addition, the capacity of the pelvic veins is increased (60%) during pregnancy, and it is likely that they do not fully return to their initial size afterwards, contributing to the formation of PCS [4,5]. It is also possible that pathological conditions such as phlebitis, history of pelvic surgery, and obesity are involved in PCS genesis.

The change of vein characteristics does not necessarily lead to pelvic pain. It is the stretching and stagnant flow of the increased volume of blood that may activate receptors in the vessel walls, which further initiate the pain signal. Therefore, not all women with these anatomic findings will develop PCS [6,7]. According to recent statistics, 60% of the population with ovarian varices may develop PCS [8,9].

One of the reasons that the diagnosis of PCS is overlooked is that no standard diagnostic criteria have been established. Some of its symptoms are common with other pathologies, and this contributes to its misdiagnosis. For example, patients may complain of dyspareunia and dysmenorrhea, which are common symptoms in endometriosis and therefore misleading for the clinical evaluation. Additionally, lower back pain, hemorrhoids, vaginal discharge, dysuria or urinary frequency and urgency along with anxiety and depression have been reported [10,11].

Underdiagnosis of PCS occurs in patients with CPP, with or without other pathological findings. In recent research, out of 293 abdomen-pelvis computed tomography (CT) scans with dilated veins, 18% were diagnosed with CPP of unclear etiology prior to the CT scan [12]. The rate of diagnosis of PCS in patients with CPP and no detectable pathologies is 10–30% [13].

CPP is a pathological condition in women, usually underestimated due to several reasons, which include the duration of pain, which should persist for at least three months until its diagnosis as chronic, and its occurrence, which could be cyclical or occasionally during certain functions (e.g., urination, menstruation). These characteristics may prevent women from asking for medical advice and prolong their period of suffering. It is well established that CPP relates to many pathological conditions, and usually the approach that the clinicians adopt for the identification of the cause is by exclusion of pelvic pathologies. This may lead to further delays in diagnosis and probably to insufficient treatment. The choice of examination (invasive/non-invasive), accompanied by physical examination, is critical.

CPP affects a patient’s life not only physically but also socially and psychologically. Generally, women will only seek professional help once the pain affects parts of their social life (e.g., work, relationships) or once they find themselves in a position that they cannot conduct regular activities without assistance (e.g., housekeeping, shopping). Under these circumstances, anxiety and depression may also form a pathological condition, requiring medical treatment. In addition, the intensity of chronic pain may change due to the pain mechanism. For example, in endometriosis, the inflammatory response activates the sensory neurons, resulting in a low threshold of pain. However, under chronic inflammatory stimulus, the sensitivity to noxious stimuli, hyperalgesia, will alter, and, consequently, any future pain will be magnified. This pain will form a memory of the unpleasant event in the cerebral cortex, which will affect the intensity of any future pain even when the pathological findings are not enough to document it [14,15,16].

Therefore, the co-existence of more than one condition related to pain in CPP is not a rare phenomenon. Other pains, pathological—either in the pelvis or outside—and psychological, may increase the threshold of pain intensity that the patient feels. This fact has promoted the belief that treating pains in total, and avoiding the neglect of any, could lead to the adequate relief of a patient’s pain [4]. This requires for a holistic approach of a patient’s experience by a multidisciplinary team of professionals. According to the National Health Service (NHS), the estimated annual cost for the treatment of CPP in 2004 was £326 million [17]. In a more recent study, the average annual total cost for the treatment of endometriosis, which is one cause of CPP, was €9579 per woman in Europe. These costs include physician visits, monitoring tests, and, in some cases, surgery and hospitalization [18].

Based on the above facts, medical management of PCS is crucial, yet it has not been proven to be effective up to an efficient level. Medical treatment targets hormonal and mechanical mechanisms [19], using nonsteroidal anti-inflammatory drugs and gonadotropin-releasing hormone (GnRH) agonists as well as medroxyprogesterone with psychotherapy. As such, first line treatment includes the use of danazol, goserelin for 1 year maximum, progestins, and oral contraceptives in combination with phlebotonics [20,21,22].

In addition, the surgical management of PCS can be based on the cause. For example, bilateral ovarian vein embolization is an option for PCS caused by gonadal and pelvic vein incompetence, and the success rate of the method could be up to 75% with low recurrence (5%) [23]. In addition, transperitoneal ligation of the ovarian vein has been shown to result in a decrease in pain [24]. This method, although it can only be applied in a limited number of refluxing veins, allows for the detection of comorbidities such as endometriosis.

## 2. Methods

A non-systematic literature review was performed using Scopus online database and Google. The combination of key words used for the research were ‘chronic pelvic pain’, ‘pelvic congestion syndrome’, and ‘laparoscopy chronic pelvic pain’. This research was conducted in order to extract information from the available documents about the success rate of laparoscopy in the diagnosis of CPP causes, especially of PCS, in comparison to other non-invasive techniques.

## 3. Results and Discussion

### 3.1. Diagnostic Tools for PCS

Non-invasive and invasive imaging techniques are the primary tool for the diagnosis of PCS, which include ultrasound (US) assessment, transabdominal duplex ultrasound, transvaginal duplex ultrasound and duplex ultrasonography, catheter-directed selective venography and intravascular ultrasound-cross-sectional CT, magnetic resonance venography (MRV), and laparoscopy [25]. Lately, venography, US, and MRI have shown improvements in the diagnosis of PCS [26].

CT scans and MRV are usually used in combination to determine the underlying cause of pelvic pain. The drawbacks of these two techniques are known and include the use of radiation and the cost. In PCS, CT scans and MRV have several issues that limit the clear visualization of pelvic veins and fail to produce an accurate imaging that will allow for the diagnosis of PCS [27]. An MRI-based technique is the use of phase-contrast velocity mapping, which can provide information on the direction and velocity of flow in different vascular channels. This method can reveal PCS after the evaluation of pelvic veins [28].

Sonography is a non-invasive method, which is usually the primary method used compared to any other screening, to exclude comorbidities [29]. Although endometriomas can be identified via an ultrasound, those of peritoneal disease may not be detected, and specialized ultrasound approaches for the detection of endometriosis have been described recently [30].

Ultrasound is used to exclude the presence of pelvic masses or uterine problems in cases of pelvic pain. The method can also reveal any anatomic elements of interest in pelvis, ovarian, and uterine changes and polycystic changes of the ovary, which are characteristics of pelvic congestion syndrome. Color Doppler and conventional B-mode may be used to monitor the retrograde flow of blood and changes in its velocity as well as any enlargement of pelvic venous channels, which can lead to the diagnosis of vein incompetence [20].

Venography is used to picture the distension of the venous channels. Patients with PCS may have congestion of flow in venous channels of ovarian, pelvic, vulvovaginal, and also thigh veins. In addition, any pelvic veins with a diameter more than 5–10 mm may also point to PCS.

### 3.2. Diagnostic Laparoscopy in the Diagnosis of PCS

Pathological findings of PCS can be identified on laparoscopies. In general, the common findings observed in women with pelvic pain via laparoscopy include pelvic inflammatory disease, ovarian cysts, pelvic adhesions, and endometriosis for which laparoscopy is the ‘gold standard’. The advantages of laparoscopy in the identification of CPP causes has been documented in cohort studies. In a sample of 487 women, almost half were diagnosed with a pathological condition (endometriosis 31%, adhesions 17%, pelvic inflammatory disease 5%) [31].

Several studies have shown that laparoscopy is able to better/accurately identify the causes of CPP in patients, including PCS, in comparison to clinical and imaging examinations [32,33,34]. In one study [34], laparoscopy was determined to be superior in up to 98% of the cases.

A recent study [35], the MEDAL study, showed that MRI scans did not provide any additional evidence of an underlying cause of the idiopathic chronic pain, and it was not proven to be cost-effective when compared with laparoscopy. More specifically, the MEDAL study included 26 hospitals in the UK, and researchers attempted to base their decision on the need to perform laparoscopy in CPP patients on imaging data. In summary, information collected on patient history, via examination and ultrasound, was enough to lead to primary estimation. In addition, the pathological conditions that were identified during laparoscopy included endometriosis, adhesions, adenomyosis, ovarian cysts, fibroids, pelvic inflammatory disease, and PCS.

Another study points out the advantages that laparoscopy may have when compare to ultrasound. According to this recent study, ultrasound screening of 150 women suffering from CPP failed to show any pelvic pathology [36]. More specifically, laparoscopy was employed after normal findings during a clinical examination and pelvic ultrasound scan. Endometriosis was found in 20% of these patients and adhesions were found in 6.7%, while, according to the authors, no pelvic disease was documented in the rest of the participants. Although the authors concluded that laparoscopy did not provide an additional benefit in regard to CPP, there are several points to consider. It is important to highlight that endometriosis was detected only by laparoscopy in a percentage that it is not small (20%). Due to this, the diagnosis of the stage of disease was possible and immediate treatment was available. These are two advantages of laparoscopy compared to imaging methods when it comes to the investigation of CPP.

Although not all conditions that cause CPP may be identified with laparoscopy or be subjected to effective treatment, for those where laparoscopy is the standard method for diagnosis, it can also offer treatment. Therefore, the use of laparoscopy is preferred for the examination of patients with CPP and no history of pelvic pathology. Laparoscopy is used for the diagnosis of CPP causes in up to 40% of cases [37]. In addition to the MEDAL study mentioned above, the history of the patient as well as physical examination and ultrasound scans should be completed in order to assist in the decision making about the need for performing laparoscopy.

Laparoscopy remains the most trusted technique for the diagnosis of endometriosis and adhesions. Moreover, laparoscopy has a sensitivity of 40% in the detection of pathologies that associate with PCS [8], and this rate is higher than US (20%) and CT scans (13%). Prominent enlarged broad ligament veins, as well as pelvic varices, can be viewed via laparoscopy [38]. It has been noted that due to the CO_2_ gas used in the method, varices maybe overlooked [39], and this is one of the reasons that laparoscopy appears to be 80%–90% negative in patients with PCS [40]. Apart from the limitations of laparoscopy, an additional limitation may also be the inexperience of the clinician in making a diagnosis of PCS. Therefore, more effort should be placed in improving the sensitivity of laparoscopy for CPP, especially towards PCS diagnosis.

Additionally, it has been mentioned elsewhere that the use of laparoscopic conscious pain mapping could potentially improve the accuracy of the method towards the diagnosis of CPP causes [41]. Unfortunately, to the best of our knowledge, there has been no recent study investigating the use of laparoscopic conscious pain mapping for the diagnosis of PCS.

The use of computational methods for image analysis in order to extract information and assist in the clinical diagnosis of pathological conditions is well known. Especially nowadays, the improvement of computational algorithms (machine learning) towards artificial intelligence has facilitated the analysis of large numbers of endoscopic images. This allows human limitations to be overcome and helps prevent human errors, resulting in high diagnostic accuracy. There are several ways in which machine learning is used for this purpose, such as the detection of various gastrointestinal abnormalities [42]. In gynecology, the use of such techniques for processing data obtained from laparoscopy is limited to image-based detection for the presence or absence of an anatomical structure. Therefore, taking advantage of machine learning for the analysis of endoscopic imaging for the diagnosis of pelvic pathologies such as endometriosis and PCS is crucial. Doing so will improve the diagnostic accuracy of laparoscopy and will hopefully reduce the time needed for the identification of CPP causes.

Lately, quite a few attempts to establish an algorithm for the diagnosis and management of PCS have been published [43,44]. However, the primary suspicion of PCS seems to depend on the clinician’s expertise, while the non-invasive imaging methods used have known limitations.

## 4. Conclusions

Based on the above, the superiority of laparoscopy in the diagnosis of PCS, for which the identification of CPP causes is usually a first approach, should be taken into consideration. Now that we, as a medical/scientific community, are looking to establish an algorithm for the diagnosis and treatment of patients with PCS, alternatives to improving laparoscopy should be considered and invested in to maximize its potential.

Laparoscopy has certain limitations, and its accuracy has been investigated. Histological examination may fail to confirm findings in laparoscopy, but the accuracy of the method is improved for a ‘negative’ finding for endometriosis. However, imaging techniques lack standardized protocols for scanning the pelvis and evaluating pelvic pain. In the case of MRI, the applied protocol depends on the clinical information and the suspected pathology, while the experience and the opinion of the radiologist has a major impact on the final diagnosis. Currently, no guidelines recommend the use of MRI as a routine procedure for the investigation of CPP.

Although there are limitations of laparoscopy, its advantages should be considered. Laparoscopy is able to overcome issues that imaging techniques have in making an accurate diagnosis. Symptoms of PCS overlap with those of other known pathological conditions of the pelvis. Even in cases where the patient’s medical history does not indicate endometriosis or other medical conditions, laparoscopy should be used as the gold standard. This will allow for the exclusion or identification of any pathology of the pelvis that relates to CPP and will help in identifying any anatomical characteristics that could suggest PCS. Subsequently, further examination with ultrasound techniques guided by laparoscopic findings should be followed. Moreover, laparoscopy has the advantage of facilitating an immediate surgical treatment as soon as a pathology of the pelvis is identified during the process. For example, in superficial peritoneal endometriosis, any imaging method would add no benefit.

More effort should be put toward developing the options available for improving the accuracy of laparoscopy in the diagnosis of pelvic pathologies. We appreciate that machine learning could make a huge impact in this regard, and we strongly advise research in this field.
